# The effect of orthotics on plantar pressure in children with infantile tibia vara (Blount's disease)

**DOI:** 10.1038/s41598-023-30066-4

**Published:** 2023-02-18

**Authors:** Enver Güven, Seyit Çıtaker, Serap Alsancak

**Affiliations:** 1 Faculty of Health Sciences, Department of Prosthetics and Orthotics, Ankara University, Ankara, 06290 Turkey; 2 Faculty of Health Sciences, Department of Physiotherapy and Rehabilitation, Gazi University, Ankara, 06490 Turkey

**Keywords:** Rehabilitation, Orthopaedics

## Abstract

Albeit some studies have revealed the effectiveness of the orthosis use in infantile tibia vara (ITV), hitherto no study has shown the effects of these orthosis on plantar pressures. This study aims to reveals the effects of orthosis on plantar pressure in infantile tibia vara. Fourteen children (mean age: 27.14 ± 5.00 months) with infantile tibia vara made up the study group and 14 healthy children (mean age: 26.42 ± 5.33 months) constituted the control group. The plantar pressure distribution was evaluated with WALKINSENSE. ITV group was evaluated before and after orthosis. The control group did not use orthosis and was evaluated once. After orthosis, it was determined that lateral foot pressure decreased, and medial foot pressure increased in the hindfoot. After orthosis, medial pressure decreased in the forefoot (*p* < 0.05). The pressure in the forefoot of the control group was significantly higher than that in the ITV group (before and after orthosis) (*p* < 0.05). After orthosis, the pressure was similar in the posterior and mid-foot sensors between the ITV group and the control group (*p*˃0.05). The orthosis can be effective in achieving the normalization of the soles pressure distribution in children with ITV.

## Introduction

Tibia vara is described as usually progressive pathology resulting in deviation of the lower extremity because of growth arrest on the medial aspect of the proximal tibial epiphysis and physis^1–4^. There are two types of deformity, namely infantile tibia vara (ITV) and adolescent tibia vara. The onset of the disease occurs before the age of 3 in the ITV, while in Adolescent tibia vara, it occurs at the age of 10 years and over. According to another classification, Tibia Vara is divided into three subtypes: infantile, juvenile, and adolescent. The juvenile type is identified to occur between 4 and 10 years old, while the adolescent type occurs at the age of 10 years and over^1,3,5,6^. For the first time, ITV was defined by Phillipp Erlacher in 1922, and following Blount's classical definition in 1937, it was named after Blount's Disease, which is widely known today. Obesity, increased genu varum and trauma are risk factors. Children with these risk factors should be under the control of a physician in the early period. Weight control in children can be beneficial in terms of prevention. Despite this, treatment methods come to the fore when ITV is seen in children. Treatment methods for ITV are mainly conservative or surgical. Orthosis and spontaneous recovery are the most emphasized methods in conservative methods^2,7–11^. The Langenskiöld classification, while orthosis is effective in stages 1 and 2, surgery is more effective in other stages. The factors that lead to the failure of conservative treatment include obesity (> 90th percentile), excessive varus angle, age (onset of treatment > 3 years), bilateral involvement, and being at stage 3 and above, based on the Langenskiöld classification^12^.

Surgery is crucial for angular and rotational changes in advanced deformities regardless of age^13^. There is not enough information in the literature about the results of orthotic approaches to ITV cases. Thus, it is not performed as widely as surgical treatments^13^. In several studies, it has been stated that orthosis shows positive developments in the knee joint^2,14,15^. Given the biomechanics of the musculoskeletal system, it is considered that ITV could affect the lower and upper segments. The musculoskeletal system is a biomechanical chain, and pathology affecting a joint can also affect the joints closest to it, below and above it. Hence, ankle and hip joints must be considered in evaluation and treatment methods. As a result of the evaluation, treatment approaches for the affected area can also be added to the treatment plan.

Pathologies in the lower limbs affect the plantar pressure distribution adversely. It can differentiate the plantar pressure distribution according to the healthy individual. It can increase the pressure in some parts of the foot, while reducing it in others. Gheluwe et al. (2005) has been shown that the subtalar joint is affected in by genu varum and genu valgum deformities, and foot pronation and supination moments during gait are also affected. The change in foot pronation and supination moments also alters the plantar pressure distribution^16,17^. Although the lower extremity is affected in individuals with ITV, there is no study on the change in plantar pressure in this group of patients. We believe that the correction of these lower extremity disorders will positively affect the development of individuals with ITV who have had a defective lower extremity structure since childhood.

This study reveals the effect of orthosis on the plantar pressure in ITV.

H0 hypothesis of the research In ITV, bracing has no effect on plantar pressure. The H1 hypothesis is that bracing effects plantar pressure in ITV.

## Methods

Approval for this study was granted by the Clinical Research Ethics Committee of Gazi University, Ankara, Turkey (ref: 2018/272). All methods were performed in accordance with the relevant guidelines and regulations.

### (Participants-procedure-data analysis)

Based on the results of the power analysis, it was determined that the number of children that must be included in the study (80% power and 5% margin of error) was 14. The legal guardians of the children who were included in the study were informed about the aim of the study and the evaluations to be made, and the “Informed Consent Form” determined by the Clinical Research Ethics Committee was read and their approval was obtained by obtaining their signatures. 14 children who were diagnosed with ITV made up the ITV group, while 14 healthy children aged between 1 and 3 years who could walk independently and without any health problems made up the control group. A total of 28 children in 2 groups were included in the study.

The inclusion criteria of the ITV group were determined to not have had lower extremity surgery, being between the ages of 1–3, being able to walk independently, not having a systemic disease or any other orthopedic problem, and being in the Langenskiöld 1st- 2nd stage. Individuals who had lower extremity surgery and could not walk independently were excluded from the study.

No application was made to the control group. The orthosis was used for 3 months by removing them for 3 h a day and remaining worn for 21 h^2^. The control group was evaluated once, while the ITV group was assessed before and following orthosis in the third month.

**Figure 1:**
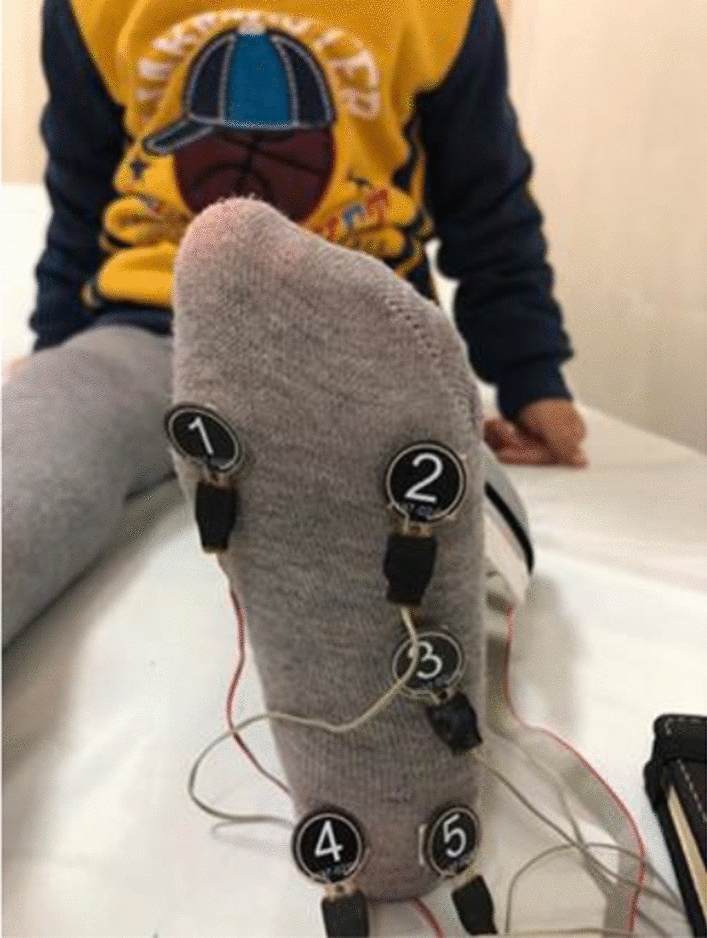
Placement of WALKINSENSE on plantar (1. Sensor:first metatarsal head, 2. Sensor: fifth metatarsal head, 3. Sensor: under the cuboid, 4. Sensor: medial of the calcaneus, 5. Sensor: lateral of the calcaneus).

The evaluation of plantar pressure in children was conducted using WALKINSENSE^18^. The WALKINSENSE plantar pressure evaluation device is a portable, non-invasive, and practical instrument to use. The device contains plantar pressure sensors, a recording apparatus, and a leg fixation band. During the measurement, the sensors were placed at 5 specified sites on the plantar foot surface of the children. Sensors were placed lateral to the calcaneus, the medial of the calcaneus, under the cuboid, the fifth metatarsal head, and under the first metatarsal head on the plantar (Fig. 1).

**Figure 2:**
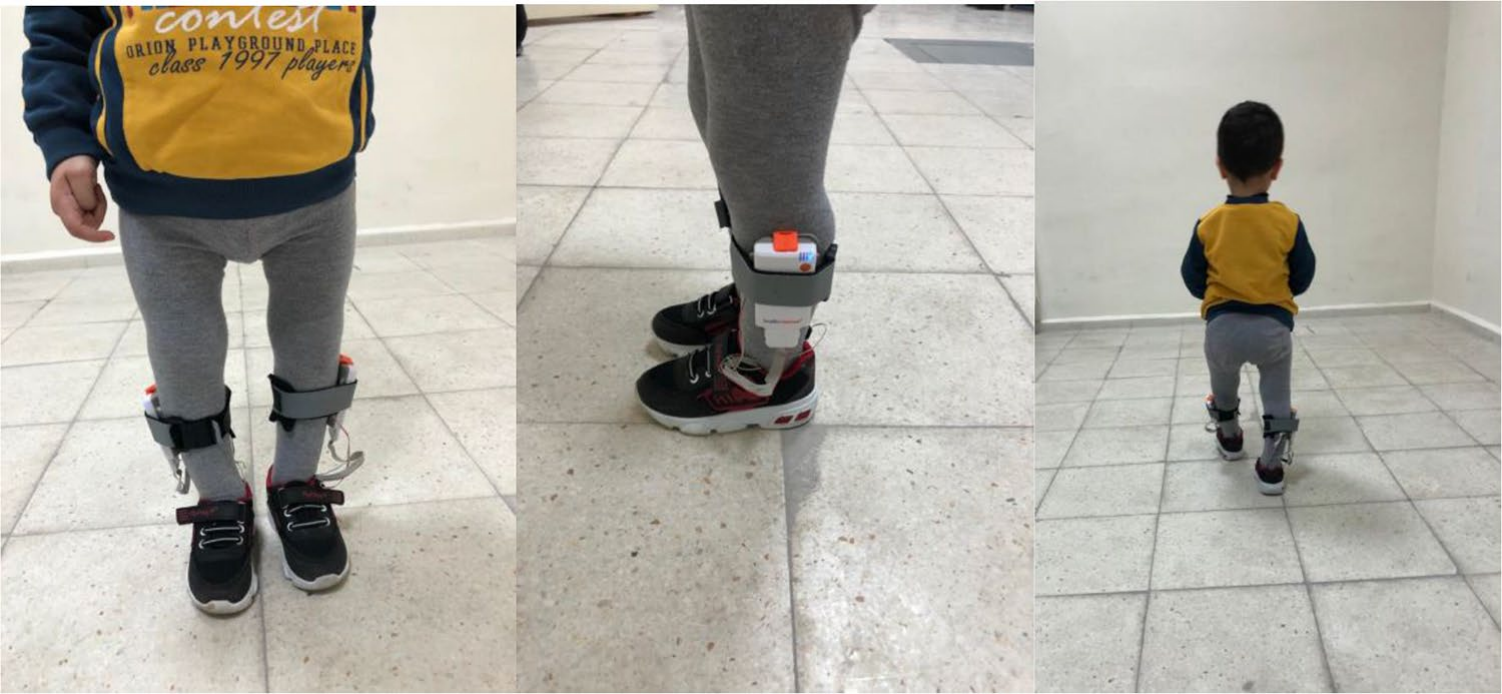
WALKINSENSE attached to the legs. (**A**) Anterior view (**B**) Lateral view (**C**) Posterior view.

The children were ensured to wear the same type of sports shoes. The anatomical regions for sensor placement were specified by the same researcher and placed on their socks. The data of each child was recorded 3 times to take at least 5–6 steps, and the data recorded in the second time was used^18^ (Fig. 2).

The sensors were connected to the Bluetooth-enabled device, which was attached to the ankle, and the device transferred the data to the software on the computer via Bluetooth. How much pressure the children applied to each sensor in the 3 phases of gait was monitored through the program. These 3 phases included heel strike, mid-stance, and push-off phase. WALKINSENSE software gave 3 phases of walking. With the information here, pressure analyzes in 3 phases were made. The pressure data transmitted from the sensors in each phase was recorded separately (kg/cm^2^). The assessment continued from the heel strike to the completion of the push-off phase. Pressure data was peak pressure.

### Orthosis

For children with ITV, type 3 knee ankle–foot orthosis (KAFO) with medial bar and ring lock, which had been used in a previous study and whose effectiveness had been shown, was used^2^. An orthosis was produced for each patient based on the lower extremity measurements of each patient.

**Figure 3:**
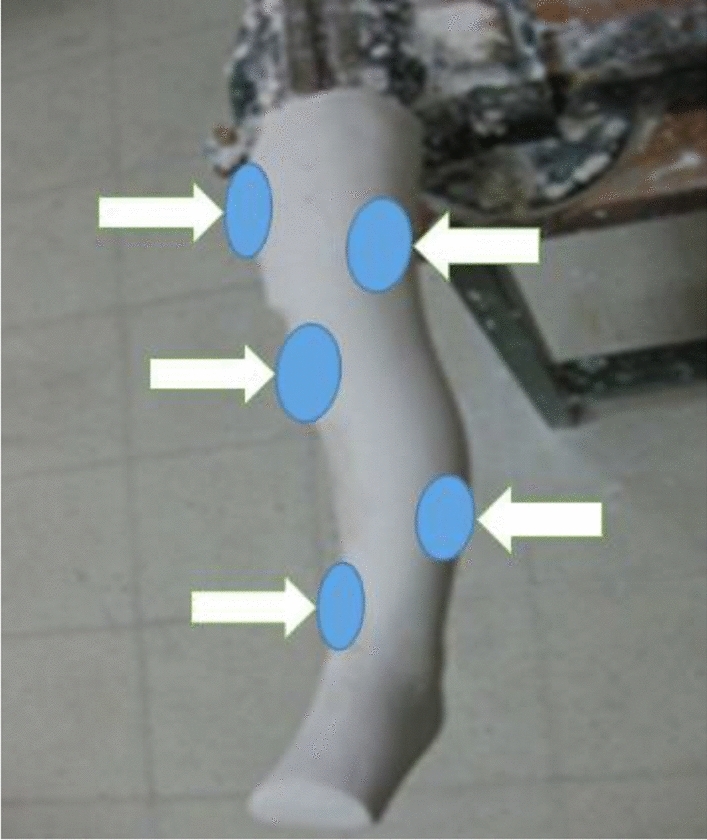
Positive model.

Three regions on the femur and two regions on the tibia were determined on the positive model for correction. Those on the tibia were the lateral region in proximal and just above the medial malleolus distally. In the femur, a counterforce region from the lateral was determined versus 2 medial forces, in the middle them (Fig. 3).

**Figure 4:**
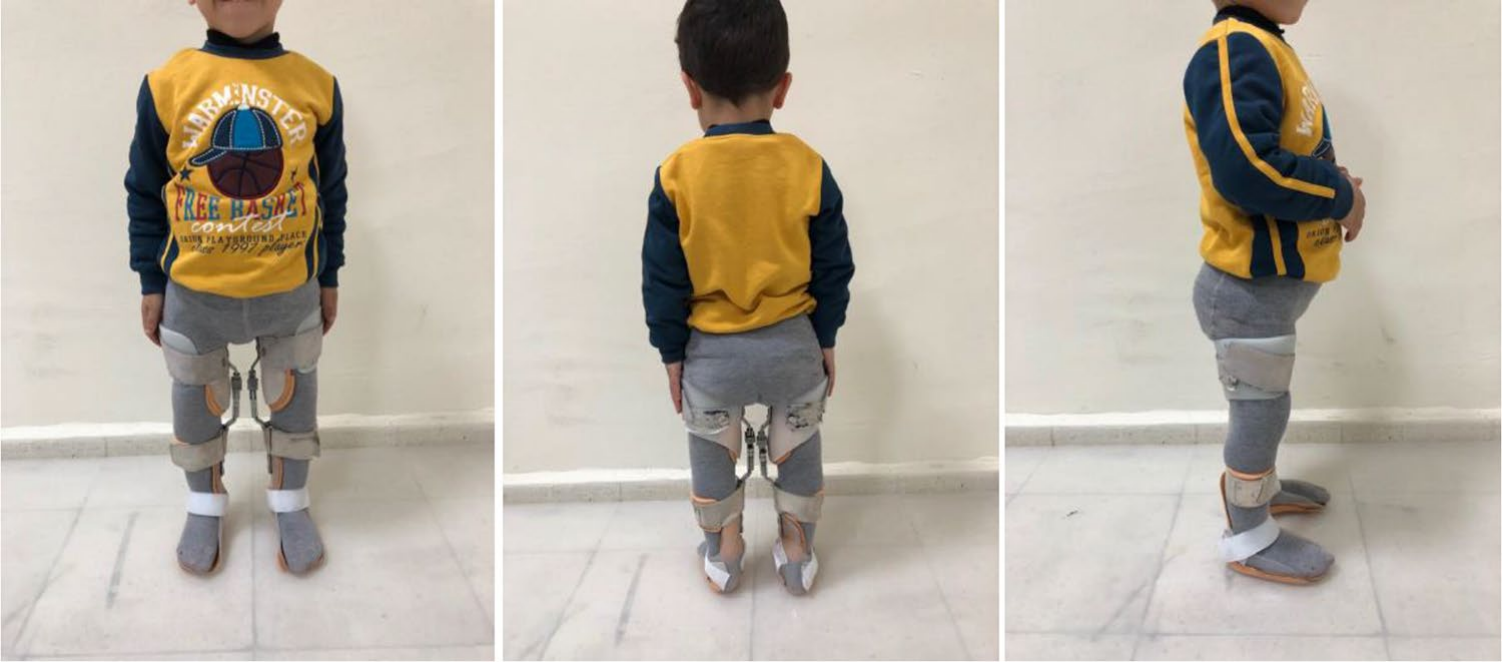
KAFO application.

In Type 3 KAFO, the force was applied over the medial malleolus of the tibia, lateral to the proximal tibia, and force was applied over the medial femoral condyle and lateral to the proximal femur. The plastic parts of the orthosis, except for the medial bar, are made of 3 mm thick polypropylene material. A ring lock was used on the medial steel bar to prevent the movement of the knee joint^2^ (Fig. 4).

### Data analysis

The data obtained were analyzed via the SPSS (Statistical Package for Social Sciences) 15 program. Mean, standard deviation, median, minimum and maximum values were used to express continuous variables (quantitative variables) that were obtained by measurement, while frequency and percentage values were used to express categorical variables (qualitative variables). The Shapiro–Wilk test was used to investigate whether the quantitative variables fit the normal distribution.

For each quantitative variable, the difference between groups for each measurement recorded at different times was analyzed using the Mann–Whitney U and independent samples *t* tests. Whether the change between measurements taken at different times within the group was significant was investigated using the generalized linear mixed model and pair-wise comparison analysis. The results were considered statistically significant at *p* < 0.05.

## Results

Of the children with ITV in the study group, 10 (71.4%) were boys and 4 (28.6%) were girls, while in the control group, 9 (64.3%) children were boys and 5 (35.7%) were girls. The groups were found to be similar in terms of sex (*χ*^2^ = 0,000 SD = 1 *p* = 1). The age, height, and body weight of both groups were similar (*p* > 0.05), whereas the BMI of the children in the ITV group was significantly higher compared to the control group (*p* < 0.05) (Table 1).

**Table 1:** Demographic characteristics of the groups.

Physical characteristics	ITV (*n* = 25)	Control (*n* = 28)	*p*
	**X ± SD**	**X ± SD**	
Sex	4/10	5/9	1.00
Age (month)	27.14 ± 5.00	26.42 ± 5.33	0.71
Height (cm)	92.78 ± 6.05	92.50 ± 4.31	0.887
Body weight (kg)	14.62 ± 3.08	12.78 ± 1.51	0.055
BMI (kg/m^2^)	16.87 ± 2.18	14.92 ± 1.24	**0.007**

*ITV* İnfantil tibia vara, *BMI* body mass ındex, *X* Mean, *SD* standard deviation.

*p < 0.05.

Of the children in the ITV group, 2 (14.3%) had the right side, 1 (7.1%) left side, and 11 (78.6%) had bilateral lower extremity involvement.

The pre-and post-orthosis data of 25 lower extremities in the ITV group were compared with the data of 28 lower extremities of 14 children in the control group.

### Comparison of pre-orthosis plantar pressures in the ITV group

In the ITV group, the pre-orthosis pressure in the lateral midfoot in the heel strike phase was determined to be significantly lower compared in the medial and lateral heel (*p* < 0.05), whereas no significant difference was determined between the lateral and medial heel (*p* > 0.05). In the mid-stance phase, it was found that the pressure in the first metatarsal was significantly higher compared to that of the medial heel, whereas the pressure in the mid-foot was significantly lower compared to that of the medial heel, and the pressure in the medial heel was significantly lower compared to that of the lateral heel (*p* < 0.05). However, no significant difference was detected between the other sensors (*p* > 0.05). In the push-off phase, it was found that the pressure in the 1st metatarsal was significantly higher compared to the 5th metatarsal and lateral mid-foot, and the pressure in the 5th metatarsal was significantly higher than the lateral mid-foot (*p* < 0.05) (Table 2).

**Table 2:** Comparison of pre-orthosis plantar pressures in the ITV group.

ITV (*n* = 25)					
		X ± SD	Med	(Min–max)	*P*
Heel strike	Lateral midfoot Medial heel	0.30 ± 0.28 0.88 ± 0.46	0.22 0.70	0.00–0.93 0.31–1.87	< 0.001
	Lateral midfoot Lateral heel	0.30 ± 0.28 0.92 ± 0.32	0.22 0.90	0.00–0.93 0.12–1.40	< 0.001
	Medial heel Lateral heel	0.88 ± 0.46 0.92 ± 0.32	0.70 0.90	0.31–1.87 0.12–1.40	1.000
Mid stance phase	1st metatarsal head 5th metatarsal head	0.79 ± 0.39 0.41 ± 0.28	0.71 0.40	0.20–1.92 0.00–1.05	0.205
	1st metatarsal head Lateral mid-foot	0.79 ± 0.39 0.53 ± 0.25	0.71 0.47	0.20–1.92 0.20–1.16	1.000
	1st metatarsal head Medial heel	0.79 ± 0.39 0.73 ± 0.41	0.71 0.58	0.20–1.92 0.00–1.86	0.005
	1st metatarsal head Lateral heel	0.79 ± 0.39 0.77 ± 0.34	0.71 0.82	0.20–1.92 0.00–1.35	1.000
	5th metatarsal head Lateral mid-foot	0.41 ± 0.28 0.53 ± 0.25	0.40 0.47	0.00–1.05 0.20–1.16	0.368
	5th metatarsal head Medial Heel	0.41 ± 0.28 0.73 ± 0.41	0.40 0.58	0.00–1.05 0.00–1.86	1.000
	5th metatarsal head Lateral heel	0.41 ± 0,28 0.77 ± 0,34	0.40 0.82	0.00–1.05 0.00–1.35	0.820
	Lateral mid-foot Medial heel	0.53 ± 0,25 0.73 ± 0,41	0.47 0.58	0.20–1.16 0.00–1.86	0.002
	Lateral mid-foot Lateral heel	0.53 ± 0,25 0.77 ± 0,34	0.47 0.82	0.20–1.16 0.00–1.35	1.000
	Medial heel Lateral heel	0.73 ± 0,41 0.77 ± 0,34	0.58 0.82	0.00–1.86 0.00–1.35	0.006
Push-off phase	1st metatarsal head 5th metatarsal head	0.91 ± 0,41 0.52 ± 0,23	0.92 0.50	0.29–1.83 0.14–1.09	< 0.001
	1st metatarsal head Lateral mid-foot	0.91 ± 0,41 0.24 ± 0,24	0.92 0.22	0.29–1.83 0.00–0.83	0.002
	5th metatarsal head Lateral mid-foot	0.52 ± 0,23 0.24 ± 0,24	0.50 0.22	0.14–1.09 0.00–0.83	< 0.001

*ITV* infantile tibia vara, *X*: mean, *SD* standard deviation, Pressure: kg/cm^2^.

*p < 0.05.

### Comparison of post-orthosis plantar pressures in the ITV group

In the ITV group, the post-orthosis pressure in the lateral mid-foot in the heel strike phase was found to be significantly lower compared in the medial and lateral heel (*p* < 0.05), whereas the pressure in the medial heel was found to be significantly higher compared to the lateral heel (*p* < 0,05). Moreover, it was observed in the mid-stance phase that the pressure in the 1st metatarsal was significantly higher than that the 5th metatarsal, whereas the pressure on the 5th metatarsal was significantly lower than that of the medial and lateral heel (*p* < 0.05). Yet, no significant difference was detected between other sensors (*p* > 0.05). In the push-off phase, it was found that the pressure in the 1st metatarsal was significantly higher compared to that of the lateral midfoot, while the pressure in the 5th metatarsal was significantly higher compared to that of the lateral midfoot (*p* < 0.05) (Table 2). However, no significant difference was determined between the pressures in the metatarsals (*p* > 0.05) (Table 3).

**Table 3:** Comparison of post-orthosis plantar pressures in the ITV group.

ITV (*n* = 25)					
		X ± SD	Med	(Min–max)	*p*
Heel strike	Lateral mid-foot Medial heel	0.11 ± 0.14 0.94 ± 0.35	0.000 0.82	0.00–0.50 0.35–1.60	< 0.001
	Lateral midfoot Lateral heel	0.11 ± 0.14 0.70 ± 0.32	0.000 0.66	0.00–0.50 0.30–1.26	0.050
	Medial heel Lateral heel	0.94 ± 0.35 0.70 ± 0.32	0.82 0.66	0.35–1.60 0.30–1.26	< 0.001
Mid Stance Phase	1st metatarsal head 5th metatarsal head	0.60 ± 0.39 0.42 ± 0.24	0.53 0.41	0.00–1.53 0.00–0.95	0.048
	1st metatarsal head Lateral mid-foot	0.60 ± 0.39 0.36 ± 0.17	0.53 0.34	0.00–1.53 0.12–0.76	0.505
	1st metatarsal head Medial Heel	0.60 ± 0.39 0.71 ± 0.32	0.53 0.69	0.00–1.53 0.22–1.36	1.000
	1st metatarsal head Lateral heel	0.60 ± 0.39 0.49 ± 0.30	0.53 0.54	0.00–1.53 0.00–1.00	1.000
	5th metatarsal head Lateral mid-foot	0.42 ± 0.24 0.36 ± 0.17	0.41 0.34	0.00–0.95 0.12–0.76	1.000
	5th metatarsal head Medial heel	0.42 ± 0.24 0.71 ± 0.32	0.41 0.69	0.00–0.95 0.22–1.36	0.008
	5th metatarsal head Lateral heel	0.42 ± 0.24 0.49 ± 0.30	0.41 0.54	0.00–0.95 0.00–1.00	0.022
	Lateral mid-foot Medial heel	0.36 ± 0.17 0.71 ± 0.32	0.34 0.69	0.12–0.76 0.22–1.36	0.199
	Lateral mid-foot Lateral heel	0.36 ± 0.17 0.49 ± 0.30	0.34 0.54	0.12–0.76 0.00–1.00	1.000
	Medial heel Lateral heel	0.71 ± 0.32 0.49 ± 0.30	0.69 0.54	0.22–1.36 0.00–1.00	1.000
Push-off Phase	1st metatarsal head 5th metatarsal head	0.71 ± 0.34 0.55 ± 0.22	0.69 0.55	0.17–1.49 0.22–1.02	0.130
	1st metatarsal head Lateral mid-foot	0.71 ± 0.34 0.20 ± 0.23	0.69 0.18	0.17–1.49 0.00–0.80	< 0.001
	5th metatarsal head Lateral mid-foot	0.55 ± 0.22 0.20 ± 0.23	0.55 0.18	0.22–1.02 0.00–0.80	< 0.001

*ITV* infantile tibia vara, *X*: Mean, *SD* standard deviation, Pressure: kg/cm^2^.

*p < 0.05.

### Comparison of pre-orthosis plantar pressures between groups

When the pre-orthosis plantar pressures of both groups were compared, it was determined that the pressure under the lateral mid-foot and lateral heel of the ITV group during the heel strike phase was significantly higher compared to the control group (*p* < 0.05); however, no significant was determined between two groups in terms of the medial heel (*p* > 0.05). Besides, in the mid-stance phase, the lateral heel and lateral mid-foot plantar pressures of the ITV group was significantly higher compared to the control group (*p* < 0.05), whereas the plantar pressure at the 1st metatarsal and 5th metatarsal was significantly lower compared to the control group (*p* < 0, 05), and no significant difference was found between the two groups in terms of plantar pressure at the medial heel (*p* > 0.05). In the push-off phase, it was determined that the pressure under the 5th metatarsal was significantly lower in the ITV group (*p* < 0.05), while no significant difference was determined between the two groups regarding the pressures under the 1st metatarsal and lateral mid-foot (*p* > 0.05) (Table 4).

**Table 4:** Comparison of pre-orthosis plantar pressures between the groups.

		ITV			Control			
		X ± SD	Med	(Min–max)	X ± SD	Med	(Min–max)	*p*
Heel strike	Lateral mid-foot	0.30 ± 0.28	0.22	0.00–0.93	0.11 ± 0.20	0.00	0.00–0.77	**0.002**
	Medial heel	0.88 ± 0.46	0.70	0.31–1.87	0.76 ± 0.61	0.57	0.10–2.09	0.134
	Lateral heel	0.92 ± 0.32	0.90	0.12–1.40	0.64 ± 0.28	0.64	0.13–1.39	**0.001**
Mid stance phase	1st metatarsal head	0.79 ± 0.39	0.71	0.20–1.92	1.02 ± 0,.8	1.00	0.42–2.21	**0.024**
	5th metatarsal head	0.41 ± 0.28	0.40	0.00–1.05	0.67 ± 0.23	0.63	0.45–1.63	**0.002**
	Lateral mid-foot	0.53 ± 0.25	0.47	0.20–1.16	0.37 ± 0.31	0.25	0.07–1.13	**0.009**
	Medial heel	0.73 ± 0.41	0.58	0.00–1.86	0.75 ± 0.63	0.52	0.10–2.22	0.363
	Lateral heel	0.77 ± 0.34	0.82	0.00–1.35	0.61 ± 0.28	0.62	0.13–1.39	**0.048**
Push-off phase	1st metatarsal head	0.91 ± 0.41	0.92	0.29–1.83	1.07 ± 0.47	1.05	0.42–2.78	0.229
	5th metatarsal head	0.52 ± 0.23	0.50	0.14–1.09	0.72 ± 0.23	0.67	0.50–1.65	**0.006**
	Lateral mid-foot	0.24 ± 0.24	0.22	0.00–0.83	0.18 ± 0.25	0.00	0.00–0.75	0.243

*ITV* infantile tibia vara, *X* mean, *SD* standard deviation, Pressure: kg/cm^2^.

*p < 0.05.

Significant values are in bold.

### Comparison of post-orthosis plantar pressures between groups


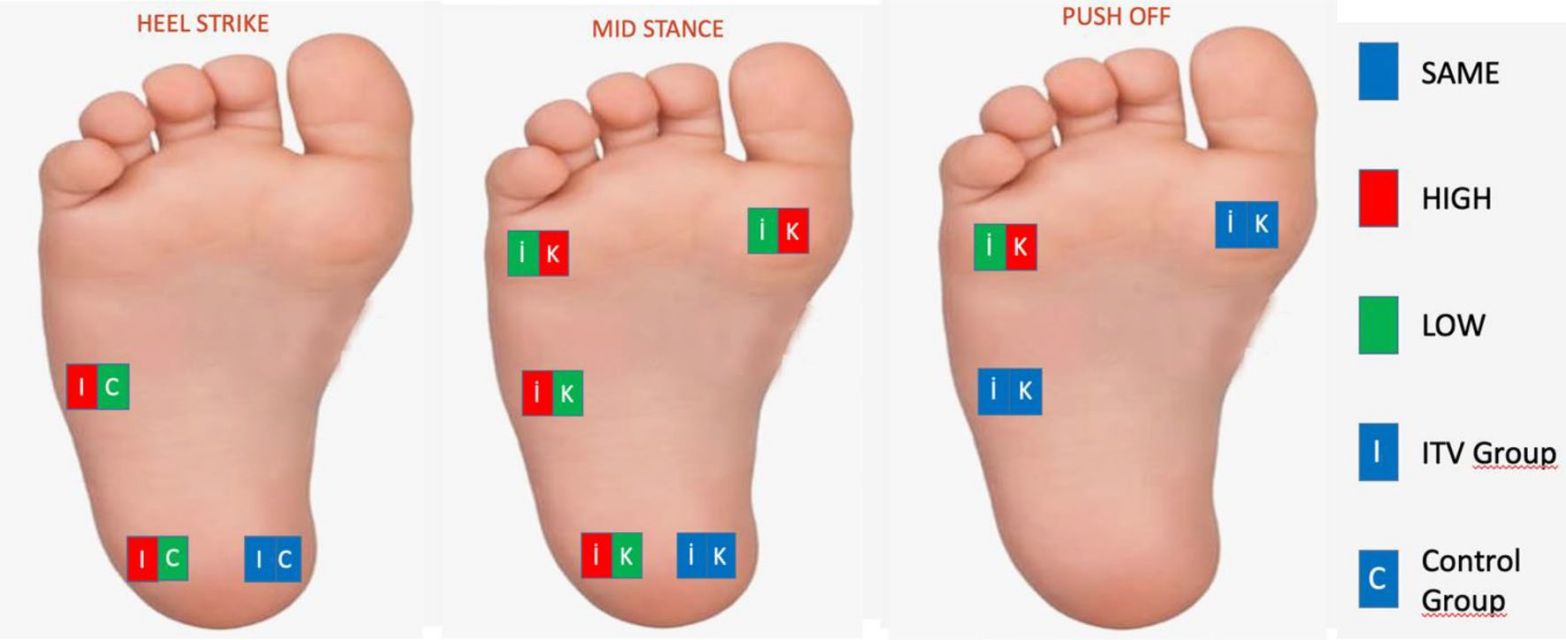


When the post-orthosis plantar pressures of the groups were compared, it was determined that the pressure under the medial heel during the heel strike phase was significantly higher in the ITV group compared to the control group (*p* < 0.05), while no significant difference was determined between the groups with respect to the lateral mid-foot and lateral heel pressures (*p* > 0.05). In the mid-stance phase, the pressure under the 1st metatarsal and the 5th metatarsal was significantly higher in the control group compared to the ITV group (*p* < 0.05), while no significant difference was detected between the two groups with respect to the other region pressures (*p* > 0.05). Besides, in the push-off phase, while the pressure under the 1st metatarsal and the 5th metatarsal was significantly higher in the control group compared to the ITV group (*p* < 0.05), no significant difference was determined between the two groups regarding the pressures under the lateral mid-foot (*p* > 0.05) (Table 5).

**Table 5:** Comparison of post-orthosis plantar pressures between groups.

		ITV			Control			
		X ± SD	Med	(Min–max)	X ± SD	Med	(Min–max)	*p*
Heel strike	Lateral mid-foot	0,11 ± 0.14	0.000	0.00–0.50	0.11 ± 0.20	0.00	0.00–0.77	0.654
	Medial heel	0.94 ± 0.35	0.82	0.35–1.60	0.76 ± 0.61	0.57	0.10–2.09	**0.028**
	Lateral heel	0.70 ± 0.32	0.66	0.30–1.26	0.64 ± 0.28	0.64	0.13–1.39	0.556
Mid stance phase	1st metatarsal head	0.60 ± 0.39	0.53	0.00–1.53	1.02 ± 0.38	1.00	0.42–2.21	** < 0.001**
	5th metatarsal head	0.42 ± 0.24	0.41	0.00–0.95	0.67 ± 0.23	0.63	0.45–1.63	** < 0.001**
	Lateral mid-foot	0.36 ± 0.17	0.34	0.12–0.76	0.37 ± 0.31	0.25	0.07–1.13	0.314
	Medial heel	0.71 ± 0.32	0.69	0.22–1.36	0.75 ± 0.63	0.52	0.10–2.22	0.310
	Lateral heel	0.49 ± 0.30	0.54	0.00–1.00	0.61 ± 0.28	0.62	0.13–1.39	0.181
Push-off Phase	1st metatarsal head	0.71 ± 0.34	0.69	0.17–1.49	1.07 ± 0.47	1.05	0.42–2.78	**0.001**
	5th metatarsal head	0.55 ± 0.22	0.55	0.22–1.02	0.72 ± 0.23	0.67	0.50–1.65	**0.011**
	Lateral midfoot	0.20 ± 0.23	0.18	0.00–0.80	0.18 ± 0.25	0.00	0.00–0.75	0.278

*ITV* infantile tibia vara, *X* mean, *SD* standard deviation, Pressure: kg/cm^2^.

*p < 0.05.

Significant values are in bold.

### Plantar pressure changes in the ITV group before and after orthosis


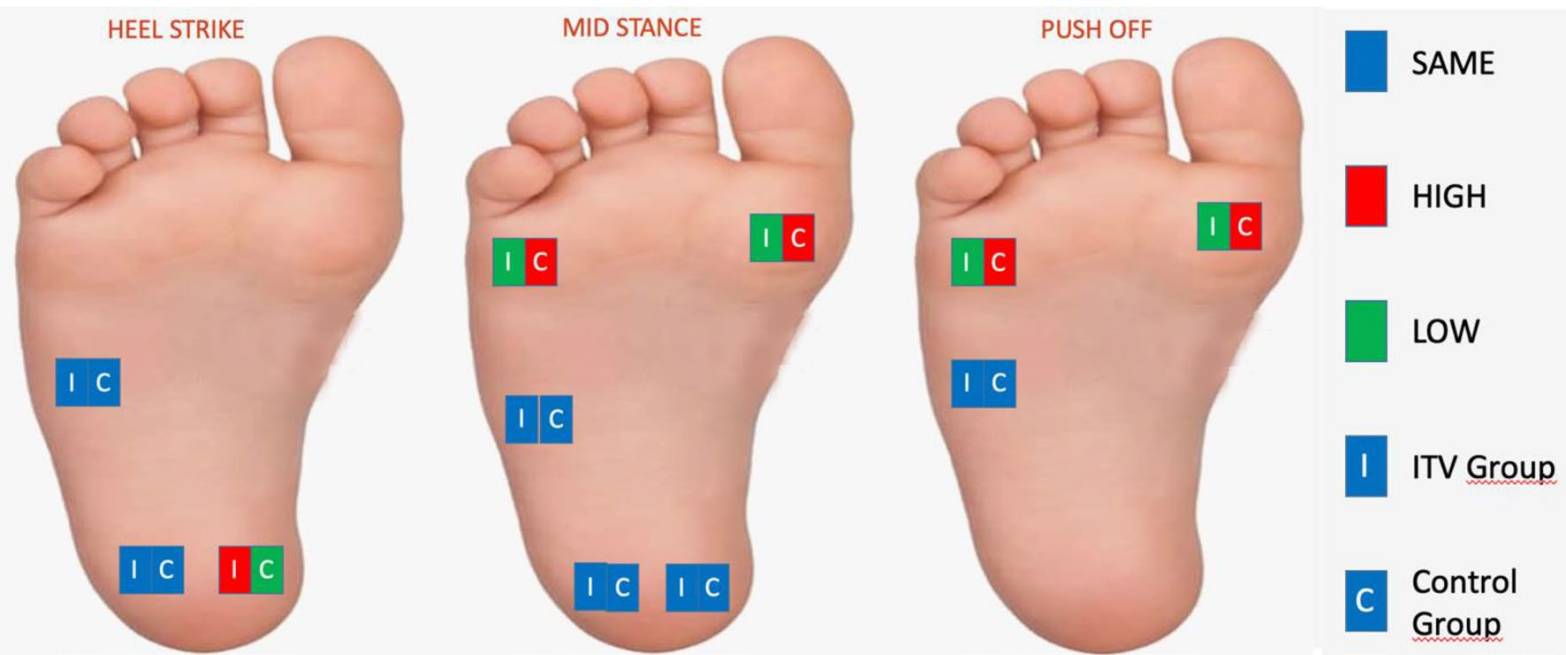


It was found that following the orthosis, the pressure on the sensors placed in the lateral heel and the lateral mid-foot decreased during the heel strike among the ITV group, whereas the pressure in the sensors placed in the heel medial increased significantly (*p* < 0.05). It was detected that in the mid-stance phase following orthosis, the pressure in the sensors placed in the 1st metatarsal head and lateral heel decreased significantly (*p* < 0.05), while no pressure change was detected in the sensors placed in the 5th metatarsal head, lateral mid-foot and medial heel (*p* > 0.05). Moreover, in the push-off phase, the pressure on the sensors placed in the 1st metatarsal head and 5th metatarsal head decreased significantly, whereas it increased significantly in the lateral mid-foot (*p* < 0.05) (Table 6).

**Table 6:** Plantar pressure change in the ITV group before and after orthosis.

ITV (*n* = 25)						
			X ± SD	Med	(Min–max)	*p*
Heel strike	Lateral midfoot	Pre-treatment Post-treatment	0.30 ± 0.28 0.11 ± 0.14	0.22 0.00	0.00–0.93 0.00–0.50	**< 0.001**
	Medial heel	Pre-treatment Post-treatment	0.88 ± 0.46 0.94 ± 0.35	0.70 0.82	0.31–1.87 0.35–1.60	**< 0.001**
	Lateral heel	Pre-treatment Post-treatment	0.92 ± 0.32 0.70 ± 0.32	0.90 0.66	0.12–1.40 0.30–1.26	**< 0.001**
Mid stance phase	1st metatarsal head	Pre-treatment Post-treatment	0.79 ± 0.39 0.60 ± 0.39	0.71 0.53	0.20–1.92 0.00–1.53	**0.001**
	5th metatarsal head	Pre-treatment Post-treatment	0.41 ± 0.28 0.42 ± 0.24	0.40 0.41	0.00–1.05 0.00–0.95	0.089
	Lateral midfoot	Pre-treatment Post-treatment	0.53 ± 0.25 0.36 ± 0.17	0.47 0.34	0.20–1.16 0.12–0.76	0.053
	Medial heel	Pre-treatment Post-treatment	0.73 ± 0.41 0.71 ± 0.32	0.58 0.69	0.00–1.86 0.22–1.36	0.331
	Lateral heel	Pre-treatment Post-treatment	0.77 ± 0.34 0.49 ± 0.30	0.82 0.54	0.00–1.35 0.00–1.00	**< 0.001**
Push-off Phase	1st metatarsal head	Pre-treatment Post-treatment	0.91 ± 0.41 0.71 ± 0.34	0.92 0.69	0.29–1.83 0.17–1.49	**0.001**
	5th metatarsal head	Pre-treatment Post-treatment	0.52 ± 0.23 0.55 ± 0.22	0.50 0.55	0.14–1.09 0.22–1.02	**< 0.001**
	Lateral midfoot	Pre-treatment Post-treatment	0.24 ± 0.24 0.20 ± 0.23	0.22 0.18	0.00–0.83 0.00–0.80	**< 0.001**

*ITV* infantile tibia vara, *X* mean, *SD* standard deviation, Pressure: kg/cm^2^.

*p < 0.05.

Significant values are in bold.


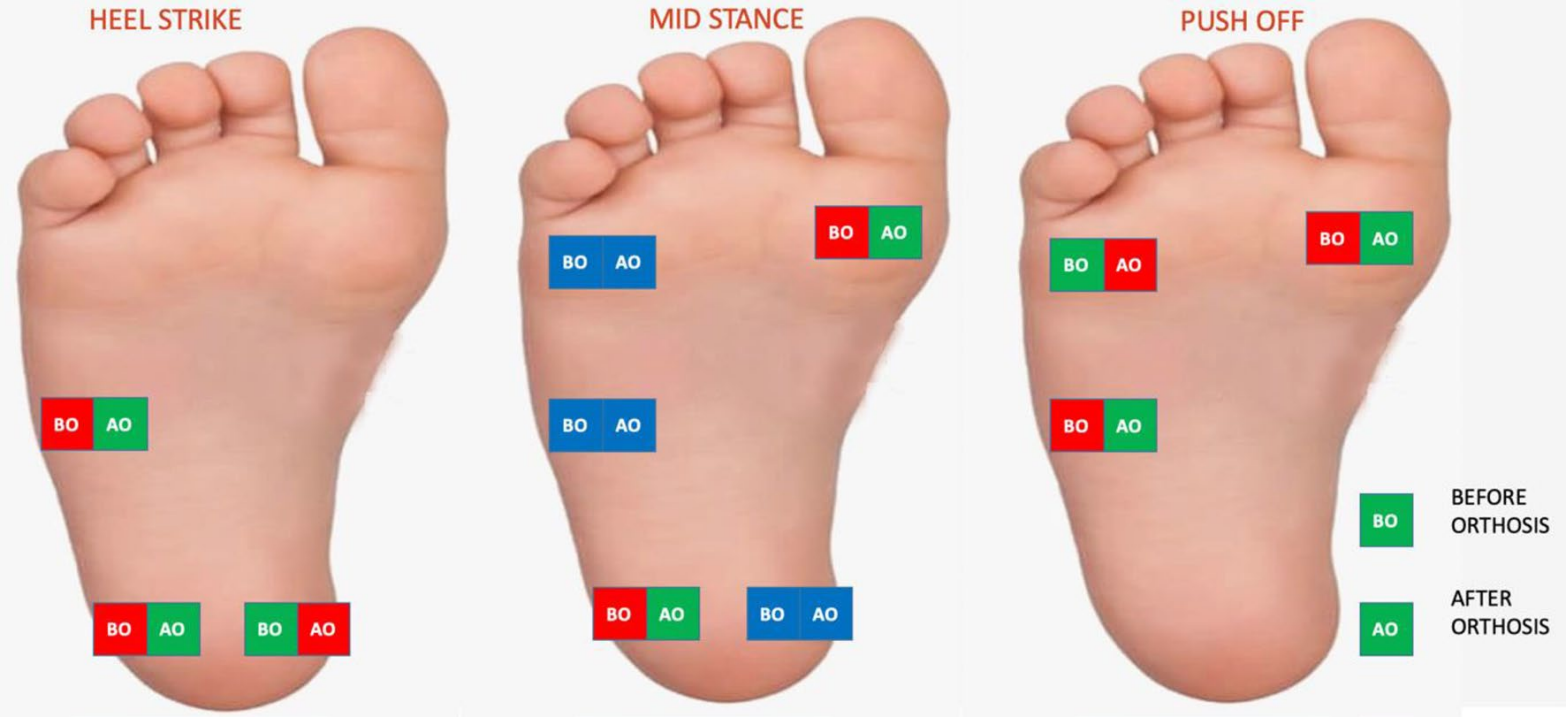


## Discussion

In the study, in which we investigated the impact of orthosis on plantar pressure in ITV, it was found that following the orthosis, the pressure in the lateral hindfoot in the ITV group decreased, while the pressure in the medial increased, and the pressure in the medial forefoot decreased. The fact that the pressure at the lateral heel and the first metatarsal head decreased was clear in all phases and comparisons. We found that orthosis in children with ITV was effective in decreasing the plantar pressure distribution closer to the level obtained in healthy children.

Some sources state that being male and obese are potent risk factors for Blount's disease, which begins at the age of 4 and later^19,20^. In our study, while no difference was determined between the two groups in terms of sex, age, height, and weight, the fact that the ITV group included more boys, and their BMI was higher than the control group supports the view that being male and being obese are strong risk factors for ITV, as revealed in the literature.

In some sources, the treatment duration of orthosis in ITV is suggested as a wider period ranging between 7 weeks and 2 years. Besides, the early period with the first signs of recovery is seen in the 3rd month^2,14,15^. Similarly, in this study, the effects of orthosis in the first period on children with ITV were investigated.

A great majority of the studies investigating the plantar pressure distribution have been conducted in adults; however, there are limited studies on children. Moreover, only one study was conducted on healthy infants^21^. It has been stated that the database on toddlers aged 1–4 is lacking^22^. Foot type and biomechanics are different in children than in adults. Hence, comparing the results with adult values might not give accurate results. Studies have been conducted mainly to investigate foot pathologies among children and adults in other age groups, particularly the plantar pressure distribution following neurological and orthopedic injury in children^21,23^. It has been suggested in the studies that increased plantar pressure is a risk factor for foot deformities and foot joint limitations^23–27^. Furthermore, it has been stated that plantar pressure is the first issue to be focused on for preventing foot deformities and their treatment^28^. It was stated in a study conducted with healthy children aged between 6 and 10 years that the highest pressure during gait was in the medial heel, lateral heel, first metatarsal head, the 5th metatarsal head, and lateral mid-foot, respectively^29^. Again, in another study conducted on juveniles, it was reported that the highest pressure during gait in healthy individuals was in the medial heel, lateral heel, the 5th metatarsal head, first metatarsal head, and lateral mid-foot, respectively^30^. In another study on children aged between10–15 years, it was revealed that the highest pressure was in the medial heel and the lowest pressure was in the lateral mid-foot among healthy individuals. Moreover, it has been shown that in individuals with planovalgus, the lowest pressure was in the lateral mid-foot^23^. Besides, it has been shown in a study on healthy infants that the highest pressure was in the heel, forefoot, and mid-foot, respectively. It has also been reported that the pressure in the medial aspect of the foot is higher than that in the lateral aspect^21^.

In a study conducted with healthy infants in the literature, the highest pressures were ranked as hindfoot, forefoot, and mid-foot, respectively^21^. Similar to the study in the literature, mid-foot pressure seems to be with the lowest pressure. Besides, it was reported in the same study that the pressure in the medial aspect of the foot is higher than that in the lateral aspect^21^. These data are also similar to the results of the study in the literature.

In a study conducted on healthy children, the highest pressure was in the medial heel^23,29^. Moreover, it has been stated that the pressure in the lateral mid-foot increased in some foot pathologies^23,30^. Furthermore, in the push-off phase, the pressure on the first metatarsal head is higher compared to the 5th metatarsal head. In other words, the pressure on the lateral aspect of the hindfoot in ITV is higher than the medial of the hindfoot. It was noticed that there are differences between the data of healthy children reported in the literature and the data of children with ITV in our study^23,29^. The pressure on the medial of the forefoot is more than the lateral of the forefoot. Based on these results, the compensation mechanism and forefoot may have increased the pressure on the first metatarsal head by moving to the valgus, when the hindfoot was in varus.

In the group with ITV, the post-orthosis pressure on the medial heel during the heel strike phase was higher than that on the lateral heel. This finding is also consistent with the data of healthy children in the literature^23,29^. In the mid-stance phase, the pressure per first metatarsal is higher than that in the the 5th metatarsal head. However, in the push-off phase, no difference was detected between the 1st metatarsal head and the 5th metatarsal head. Following orthosis, while the pressure on the medial of the heel increased, the pressure difference between the 1st metatarsal head and the 5th metatarsal head was balanced during the push-off phase. Considering these results, it is seen that thanks to the orthosis, hindfoot varus moved to valgus, and pronation in the forefoot decreased. The movement of the varus in the hindfoot to the valgus suggests that it may affect the upper segment of the knee joint; hence, the genu valgum may return to its normal limits.

In the studies conducted on healthy children, the lateral mid-foot was the region with the lowest pressure^21,23,29^. The heel lateral was the region with the lower pressure data compared with the medial heel. In our study, it was found that the difference between healthy children and children with ITV is consistent with these studies. During the mid-stance phase, the pressure in the ITV group is lower at the 1st and 5th metatarsal heads. Moreover, during the push-off phase, the pressure at the 5th metatarsal head was lower in the ITV group. It is seen that there is more pressure in the hindfoot and lateral to the mid-foot and less pressure in the lateral forefoot in ITV. These results suggest that the hindfoot moved to varus, including the mid-foot, the pressure on the forefoot is reduced, but the valgus compensation mechanism is formed in the forefoot.

After orthosis, the pressure in the medial heel during the heel strike phase is higher in the ITV group compared in the control group. In other words, unlike orthosis, the load shifted from lateral to medial. We have already mentioned above that the highest pressure was found in the heel medial in the previous studies^23,29^. In the mid-stance phase, the pressure under the 1st metatarsal and 5th metatarsal is lower in the ITV group, similar to the pre-orthosis results. However, it is noticed that there is no significant difference in the regions where other sensors are attached.

It can be seen that the post-orthosis plantar pressure distribution is similar to that of the control group. It can be said that the plantar pressure distribution of the ITV changes with the effect of the orthosis and this distribution approaches normal values.

Comparing the ITV group before and after orthosis, the pressure in the lateral heel and lateral mid-foot decreased, whereas the pressure in the medial heel increased during heel strike. During the mid-stance phase, the pressure in the first metatarsal and the lateral heel decreased. In the push-off phase, the pressure in the 1st metatarsal and 5th metatarsal decreased, whereas it increased in the lateral mid-foot. It is clear in all phases and comparisons that the pressure has decreased at the lateral heel and the first metatarsal head.

These data also support the view that varus in the hindfoot and pronation in the forefoot has been reduced.

Considering the results, varus in the tibia and knee affects the foot. Based on our results, it is considered that the hindfoot moved to the varus and the forefoot moved to pronation in children with ITV. Orthosis and plantar pressure data in the ITV group were similar to those of healthy children.

Another remarkable finding is that the pressure on the forefoot before and after orthosis is higher among ITV. ITV causes changes in the anteroposterior pressure distribution and leads to differences in the mediolateral pressure distribution of the foot. In the ITV group, the pressure per 1st metatarsal in the forefoot decreased following orthosis, yet no significant difference was observed in the pressure on the 5th metatarsal head. In the ITV group, after orthosis, the load on the lateral heel in the hindfoot decreased, after whereas the load on the medial heel increased. According to the early results of orthosis, the pressure on the forefoot in ITV did not reach the values of healthy children. In future studies on this subject, it is important to increase plantar pressure analysis studies both in pathology and in healthy age groups.

There were studies in the literature on spontaneous recovery, and it has been reported that an average of 1 of 3 patients had spontaneous recovery^10^. Lower spontaneous recovery rates and these patients seeking surgery in the absence of recovery are risks of waiting for spontaneous recovery. However, the success of orthosis in treatment is higher with the identification of eligible patients in the early stages^2,14,15,31^. For these reasons, a spontaneous recovery group was not established in our study, and the early outcomes of orthosis supported its effectiveness in treatment.

In these studies, the results of orthosis on the knee joint are given. No evaluation was made on foot, ankle and plantar pressure after orthosis. It would be beneficial to repeat the study with larger sample groups in future studies^32,33^.

Conducting the study, which looked at the relationship between walking speed and peak pressure gradient in healthy individuals, also in individuals with ITV will reveal valuable results^34^.

The fact that the ages and stages of the children in the control group matched with the study group and the comparison of the results with the control group are the strengths of our study.

### Limitations

The limitation of our study is that the control group was not assessed together with the ITV group after 3 months. Besides, the lower incidence of the disease and late application for treatment are among the factors that reduce the number of children. The lack of a real control group and follow-up data in the healthy group is a limitation of our study.

## Conclusion

The following conclusions were reached because of the study in which we examined the impact of orthosis in ITV on plantar pressure.

- Varus in the tibia and knee impacts the foot in children with ITV. The hindfoot moves to the varus, whereas the forefoot moves to the pronation. Orthosis in children with ITV reduces the varus in the hind leg and the pronation of the forefoot.
- In children with ITV, the pressure on the forefoot is lower than that in healthy children both before and after orthosis. ITV leads to changes in the anteroposterior pressure distribution and results in differences in the mediolateral pressure distribution of the foot.
- Orthosis does not alter the anteroposterior pressure distribution in children with ITV.
- In children with ITT, the plantar pressure distribution changes following orthosis and approaches normal values.

When compared with the results of healthy infants, it is noticed that orthosis in ITV has favorable effects on the plantar pressure distribution from the early period, yet there are differences in the forefoot pressure distribution. We believe that revealing the long-term effects of orthosis through further studies could provide more information on the effectiveness of the treatment.

## Data Availability

All data generated or analyzed during this study are included in this published article.
